# Incidence of New-Onset Hypertension in Cancer Patients: A Retrospective Cohort Study

**DOI:** 10.1155/2013/379252

**Published:** 2013-12-16

**Authors:** Kathy H. Fraeman, Beth L. Nordstrom, Weixiu Luo, Sarah H. Landis, Sumitra Shantakumar

**Affiliations:** ^1^Health Economics and Epidemiology, Evidera, 7101 Wisconsin Avenue, Suite 600, Bethesda, MD 20814, USA; ^2^Evidera, 430 Bedford Street, Suite 300, Lexington, MA 02420, USA; ^3^Worldwide Epidemiology, GlaxoSmithKline, 1-3 Iron Bridge Road, Stockley Park West, Uxbridge, Middx UB11 1BT, UK; ^4^Worldwide Epidemiology, GlaxoSmithKline, Five Moore Drive, P.O. Box 13398, Research Triangle Park, NC 27709, USA

## Abstract

This retrospective cohort study was conducted to estimate incidence rates of new-onset hypertension in adult cancer patients identified from the Varian Medical Oncology outpatient database. Incidence rates of increasing levels of hypertension severity were calculated overall and for periods of chemotherapy exposure and nonexposure. Cox models sought predictors of new-onset hypertension severity among baseline and chemotherapy exposure variables. New-onset hypertension was observed in about one-third of 25,090 patients with various cancer types. The incidence rates (IR) of severe and crisis-level hypertension, respectively, were the highest in patients with gastric (18.5 cases per 100 person-years (PY), 5.6 per 100 PY) and ovarian cancer (20.2 per 100 PY, 4.8 per 100 PY). The highest IR of moderate hypertension was observed in patients with renal cancer (46.7 per 100 PY). Across all cancers, chemotherapy exposure was associated with a 2–3.5-fold increase in risk of any degree of hypertension compared to periods of no chemotherapy; higher hypertension levels showed greater variability in relative risks by type and line of therapy but indicated an overall increase associated with chemotherapy exposure. These results help to elucidate the factors influencing HTN among cancer patients and the incidence of HTN relative to chemotherapy exposure.

## 1. Introduction

Hypertension (HTN) is the most common cardiovascular disease; approximately 28.5% of United States (US) adults are hypertensive [1, 2]. The coexistence of HTN and cancer among patients is common, and both HTN and cancer incidence rates have been reported to increase with age [3–7]. Hypertension is reported regularly in cancer patients, with estimates of HTN approaching or eclipsing 40% in cancer populations [4, 8–13]. The reported HTN prevalence is even higher in elderly and black cancer patients [4, 8, 11].

New-onset HTN has been reported as an adverse event for numerous cancer therapies, with risk estimates regularly approaching 10% and in certain instances as high as 36% [14–17]. These new-onset HTN rates vary greatly by cancer treatment type and dose, and increased risk of developing high-grade HTN at severe or crisis levels has been observed with more recent cancer therapies, such as those that target vascular endothelial growth factor (VEGF), known as anti-VEGF agents [18, 19]. The mechanism(s) by which different chemotherapy agents induce HTN is not fully understood. Potential mechanisms include vascular rarefaction (decrease in microvessel density), decreased sex hormones leading to impaired vasodilator and potentiated vasoconstrictor effects, and endothelial dysfunction causing an interference with nitric oxide (NO) signaling and thereby an increase in oxidative stress [15, 16, 20].

As HTN is a risk factor for coronary heart disease, stroke, heart failure, and end-stage renal disease [20], an improved understanding of HTN prior to starting chemotherapy, new-onset HTN during and after chemotherapy, and factors influencing HTN among cancer patients is paramount. Prior studies have focused on the prevalence of HTN in cancer patients rather than incidence [4, 8–13], but the incidence of new-onset HTN is an important clinical outcome that requires investigation. Using an electronic medical record (EMR) database of outpatient oncology practices, the present study uses sequential blood pressure (BP) measurements to identify new-onset HTN of different severity levels, allowing an estimation of incidence of HTN in cancer patients with solid tumors that are highly vascularized and/or are dependent on VEGF stimulation for growth.

## 2. Patients and Methods

### 2.1. Data Source

This retrospective cohort study was conducted using the Varian Medical Oncology EMR database, which contained data on more than 185,000 cancer patients at the time of data extraction for this study. The database included information from 18 outpatient oncology practices in 15 states across the United States. During each patient visit to the clinic, the clinic staff enters data into the database about the visit, including diagnoses, treatments, and other relevant information. Treatment data include orders or prescriptions for antineoplastic agents, with specific information such as dose and route, as well as duration of supply of oral medications and amount and timing of drugs administered in the clinic. Blood pressure (BP) measurement data are entered separately for systolic Blood pressure (SBP) and diastolic blood pressure (DBP) and include the date of the test and measurement result in mm Hg. The data used for the present study were deidentified, as required by the Health Insurance Portability and Accountability Act (HIPAA).

### 2.2. Study Population

The study cohort included adult patients (age ≥18 years) with any of 11 cancer types of interest—breast, lung, colorectal, head and neck, gastric, ovarian, cervical, renal, melanoma, prostate, and connective and other soft tissue—diagnosed between January 1, 2000, and April 30, 2008. The International Classification of Diseases, 9th Revision (ICD-9) diagnosis codes for these cancer types are given in Table 1. Patients with multiple primary cancer diagnoses were excluded from the study as were patients with unknown age or gender.

The index date was defined as the first date of entry of a qualifying ICD-9 cancer diagnosis code. The period from 30 days prior to the index date through the index date was defined as the baseline period, and the period from the day after the index date to the last recorded BP measurement on or before the study end date of July 31, 2008, was defined as the follow-up period. To be eligible for inclusion in the study, patients were required to be normotensive or prehypertensive at baseline (i.e., SBP ≤ 150 mm Hg and DBP ≤ 100 mm Hg) and to have at least 1 BP measurement during the follow-up period.

### 2.3. Outcome Definition and Measures

The study outcome was new-onset hypertension separated into three mutually exclusive categories of moderate, severe, and crisis-level hypertension. The BP ranges for each hypertension outcome category were based on definitions provided by the Seventh Report of the Joint National Committee of Prevention, Detection, Evaluation, and Treatment of High Blood Pressure [21].

- Moderate hypertension was defined as either of the following: (1) follow-up SBP increased to >150–160 mm Hg, or (2) follow-up DBP increased to >100–110 mm Hg.
- Severe hypertension was defined as either of the following: (1) follow-up SBP increased to >160–180 mm Hg, or (2) follow-up DBP increased to >110–120 mm Hg.
- Crisis-level hypertension was defined as either of the following: (1) follow-up SBP increased to >180 mm Hg, or (2) follow-up DBP increased to >120 mm Hg.

The time to first occurrence of each level of hypertension was calculated as number of days from the index date to the date of the first hypertensive BP measurement at that level. The same patient was eligible for each of the three hypertension outcomes.

### 2.4. Chemotherapy Exposure Definition and Measures

For each eligible patient, all person-time of followup after the index date was divided into periods of time with and without chemotherapy exposure. Chemotherapy exposure comprised the periods of one or more chemotherapy regimens consisting of combinations of cytotoxic and/or targeted chemotherapy agents administered orally or through IV/injection. The time periods outside chemotherapy regimens were considered the periods with no chemotherapy exposure.

All chemotherapy agents administered through IV or injection were assigned an effective duration of 30 days. The duration of oral chemotherapy agents was determined by a combination of available variables for prescribed days' supply, dispensed quantity, administration frequency, and number of refills.

The specific agents in a chemotherapy regimen were determined by all cytotoxic or targeted chemotherapy agents, either prescribed orally or administered through IV or injection at the clinic, during the first 8 days of the regimen. The chemotherapy regimen continued while only these agents were administered. If a new drug was given on day 9 or later within a regimen, the first regimen was considered to have ended and a new regimen begun. This new regimen consisted of all chemotherapy agents administered from the start date of the first drug through the next 8 days. This cycle of assigning chemotherapy agents to regimens continued until all chemotherapy days were assigned to a regimen. Patients were allowed to stop receiving a chemotherapy agent without ending the regimen, as long as at least 1 agent in the regimen continued without the addition of any new chemotherapy agents. Substitution of a chemotherapy agent with another in the same class but with generally higher tolerability (substituting carboplatin for cisplatin or epirubicin for doxorubicin) did not start a new chemotherapy regimen. The reverse of switching to the less tolerable agent was considered a new regimen.

Gaps of up to 14 days between the end of a chemotherapy administration and the start of the next administration within a regimen were ignored. If a gap between chemotherapy administrations was 15 to 89 days, then the gap was considered to be an off-chemotherapy break in the regimen, but the same line of therapy resumed when the chemotherapy administrations resumed. If a gap lasted for 90 or more days, then restarting the same chemotherapy agents was considered to be a new line of therapy.

### 2.5. Covariate Definition and Measures

Covariates included patient demographics (age and gender), year of diagnosis, cancer type, geographic region, clinic type, health insurance type, line of chemotherapy, and type of chemotherapy regimen. Patient age was calculated as the number of years from the year of birth to index date and categorized into groups of 18–<30, 30–<50, 50–<65, and ≥65 years. The year of the first qualifying cancer diagnosis was categorized into intervals of 2000–2002, 2003–2005, and 2006–2008. Cancer type was determined by ICD-9 codes and was categorized into breast, lung, colorectal, head and neck, gastric, ovarian, cervical, renal, melanoma, prostate, and soft tissue sarcoma.

### 2.6. Data Analysis

All data management and analyses were conducted using SAS version 9.2. Incidence rates of each level of hypertension severity were calculated both overall and separated into the periods of chemotherapy exposure and no chemotherapy exposure. The overall incidence rates were computed as the number of patients with each level of new-onset hypertension divided by the sum of all follow-up person-time through the latest available BP measurement, ending at the date of event for the patients who developed each level of hypertension severity.

Time to first occurrence of each level of hypertension severity was calculated for each patient and summarized with the median, mean, and standard deviation (SD) among patients who developed hypertension. The BP measurement at the first occurrence of each level of hypertension severity was summarized with the median and mean (SD). The incidence rates of hypertension severity during chemotherapy were calculated as the number of patients with each new-onset hypertension level divided by the sum of all follow-up intervals of time on chemotherapy in which there were BP measurements. Follow-up time for each on-chemotherapy interval was truncated at the last BP measurement during the interval. For patients with each new-onset hypertension level during followup, follow-up time was truncated as of the date of the event. Parallel rules for each level of hypertension severity were used to calculate the incidence rates during the intervals without chemotherapy exposure.

Cox proportional hazards regression analyses were performed to identify predictors of each level of new-onset hypertension among the baseline characteristics, including patient age and gender, year of index date, cancer type, geographic region, clinic type, health insurance type, and baseline BP measurement. For the moderate and severe hypertension models, to avoid censoring patients whose first elevated BP fell into one of the more severe categories, the outcomes were modeled as nonmutually exclusive outcomes of (1) moderate, severe, or crisis-level hypertension and (2) severe or crisis-level hypertension. Chemotherapy exposure, including both line and type of chemotherapy, was entered into the models as time-varying covariates, with no chemotherapy exposure used as the reference level.

## 3. Results

### 3.1. Study Population

A total of 38,940 adult cancer patients had an initial diagnosis of 1 of the 11 cancer types of interest between January 1, 2000, and April 30, 2008. Of these patients, 30,682 had a BP measurement at baseline; 25,090 had only normal BP measurements during the baseline period and at least 1 BP measurement during the follow-up period, hence qualifying for the study.

Of the 25,090 patients included in the analyses (Table 2), 64.5% were female, and the mean age was 61 years. The most common cancer type was breast (36.1%), and most of the patients (88.6%) had their first cancer diagnosis between 2003 and 2008. The majority of patients (70.7%) were classified as having systolic BP of 120–150 mm Hg and/or diastolic BP of 80–100 mm Hg.

### 3.2. Time to First Occurrence of Hypertension

The times to first occurrence of a hypertensive BP measurement for each level of hypertension severity are given in Table 3. The median number of days to the first moderate hypertension event was 96 days, increasing to 122 days for severe hypertension and 183 days for crisis-level hypertension. Elevated SBP was more common and occurred earlier than elevations in DBP for each level of hypertension severity. The mean SBP level at first occurrence of moderate hypertension was 155 mm Hg (SD: 3.1), and the mean DBP level at the first occurrence of moderate hypertension was 104 mm Hg (SD: 2.9). These levels increased to 168 mm Hg (SD: 5.5) SBP and 114 mm Hg (SD: 3.0) DBP for severe hypertension and 188 mm Hg (SD: 7.6) SBP and 131 mm Hg (SD: 10.8) DBP for crisis-level hypertension.

### 3.3. Incidence of Hypertension

Across all cancer types combined, the incidence rate of new-onset hypertension of any severity was 32.16 cases per 100 person-years (95% confidence interval CI: 22.02–45.37) (results not shown).  Table 4 provides incidence rates of each level of hypertension severity for all patients and separately by tumor type for the entire follow-up period, for the periods of chemotherapy exposure, and for the periods when patients were not exposed to chemotherapy. Moderate hypertension was a fairly common occurrence among these cancer patients, with an overall incidence rate of 27.26 cases per 100 person-years (18.00–39.59). Rates decreased as the level of hypertension increased, with an overall incidence rate of 12.36 cases per 100 person-years (6.46–21.42) for severe hypertension and 2.79 cases per 100 person-years (0.53–8.45) for crisis-level hypertension.

For all levels of severity, the incidence of hypertension was considerably higher during periods of chemotherapy exposure than while patients were not exposed to chemotherapy. The overall incidence rates of moderate hypertension during chemotherapy and during periods of no chemotherapy were 90.07 (CI: 72.43–110.70) and 20.89 (12.92–31.97) cases per 100 PY, respectively. A similar pattern of higher rates during chemotherapy exposure was seen for severe and crisis-level hypertension, although the rates were progressively lower in the latter 2 groups, with rates in the periods during and without chemotherapy exposure, respectively, of 40.21 and 9.64 cases per 100 PY for severe hypertension and 8.98 and 2.09 cases per 100 person-years for crisis-level hypertension.

One caveat to these results is that the monitoring of the patients' BP occurred more frequently during the periods when they were being treated with chemotherapy, with means of 34 measurements per person-year on chemotherapy and 6 per person-year off chemotherapy. This difference in the number of available measurements during the different periods of chemotherapy exposure allows greater opportunity to find an elevation during chemotherapy exposure.

Rates of each level of hypertension varied considerably across tumor types (Table 4). Patients with melanoma, breast cancer, and connective and other softtissue cancers had the lowest incidence of hypertension at each of the severity levels. Moderate hypertension was most commonly seen in renal and lung cancers; severe in ovarian, gastric, prostate, and lung cancers; and crisis-level hypertension in gastric, ovarian, lung, and colorectal cancers.

Within each tumor type, the finding of higher rates of each level of hypertension associated with periods of chemotherapy use compared to nonuse held constant. For the most severe (crisis-level) hypertension outcome, the highest rate observed was 19.54 (11.86–30.33) cases per 100 PY among renal cancer patients during chemotherapy exposure, and the lowest rate of this outcome was 1.26 (0.06–6.01) cases per 100 PY in connective and other softtissue cancers during periods of no chemotherapy use.

### 3.4. Predictors of Hypertension

Results of separate Cox proportional hazards models of moderate or higher, severe or higher, and crisis-level hypertension revealed an increased risk in all models associated with increasing age and with baseline prehypertension (Table 5). Many differences were seen by cancer type, with the highest risk of moderate or higher hypertension in patients with renal, cervical, and prostate cancers and the lowest risk in breast cancer and malignant melanoma. For severe or crisis-level hypertension and for crisis-level hypertension only, the highest risk was found in patients with gastric cancer and the lowest risk in patients with connective and other soft tissue and breast cancers.

As seen in the incidence rate data, the risk of hypertension was considerably higher among patients on chemotherapy in all models. These results were found with any type of chemotherapy (cytotoxic, targeted, or combination) and in all lines of chemotherapy, with hazard ratios (HR) ranging from 2.2 to 3.5 for moderate or higher hypertension, 1.6–8.0 for severe or higher hypertension, and 1.2–6.7 for crisis-level hypertension, although these risks were not statistically significant for the severe and crisis-level hypertension models.

## 4. Discussion

The present analysis of outpatient oncology EMR data examined a cohort of patients with any of 11 solid tumors who were not hypertensive at the time of their first cancer diagnosis. We found incident hypertension to have a median time to onset of 96 days after the first cancer diagnosis and an overall incidence of 27 cases per 100 PY for moderate hypertension, 122 days to onset and 12 cases per 100 PY for severe hypertension, and 183 days to onset and 3 cases per 100 PY for crisis-level hypertension. Although the incidence rates of severe and crisis-level hypertension are lower than the rate of moderate hypertension for all cancer types, these lower rates are of more clinical concern because of the associated increased risks of coronary heart disease, stroke, heart failure, and end-stage renal disease.

All levels of hypertension severity appeared to be far more frequent during periods of chemotherapy use than nonuse for all cancer types. However, blood pressure was monitored far more often during chemotherapy, and the extent to which the more frequent monitoring may have biased results in favor of finding higher rates of hypertension with chemotherapy treatment is difficult to ascertain. The relationship between cancer treatment and hypertension as an adverse event has been well documented for a variety of agents [15, 16]. It is therefore likely that the increased rates seen here during chemotherapy are not merely a statistical artifact resulting from the more frequent monitoring, although the magnitude of the difference may be overestimated in the present study. Conversely, patients exhibiting increasing BP during chemotherapy may have been started on an antihypertensive medication or, for those already on an antihypertensive, had the dose increased. Antihypertensive use is not reliably recorded in this oncology database, and so changing patterns of use during chemotherapy exposure and after the end of a course of chemotherapy cannot be determined. Continued use of antihypertensives after the end of chemotherapy could lead to decreased BP relative to baseline, whereas discontinuing the antihypertensive may be associated with a reduced need for frequent BP monitoring.

Patients with breast cancer and malignant melanoma had the lowest incidence of hypertension at all severity levels compared to patients with other cancer types. For the more severe levels of hypertension, gastric and ovarian cancers were associated with the highest rates. These variations by cancer type might be related to the different cancer types and their progression, the specific treatments used for each cancer, and/or other risk factors that are associated with both the specific tumor type and hypertension.

There are several limitations to the present study that should be noted. As discussed above, the monitoring of blood pressure was not consistent and has in our analyses been found to be biased toward more monitoring during chemotherapy exposure, which may lead to underestimation of hypertension during the periods without chemotherapy exposure. Any blood pressure measurements that were taken outside the oncology clinic would not appear in the EMR database, although the treating oncologist may have been aware of those outside results, and they may have impacted patient care. For patients who had controlled hypertension at baseline through the use of antihypertensives, the incidence of hypertension during followup reflects breakthrough or worsening hypertension rather than new-onset hypertension.

In this study, we used a single BP measurement to capture a hypertension event for each level of hypertension severity. Patients who had a single high BP due to the “white-coat effect” would be misclassified as hypertensive patients. However, we used relatively high BP thresholds to define even the lowest level of BP severity. Patients with a single BP measurement higher than 150 mm Hg SBP or 100 mm Hg DBP are likely to be genuinely hypertensive and not experiencing a brief increase in BP. Even if a few cases of white-coat hypertension were included in the analyses, it is unlikely that this would introduce any systematic bias.

This study looked at the natural history of cancer and the associated incidence of hypertension in various cancer types during periods of cancer treatment. The risk of hypertension associated with specific cancer treatments or supportive therapies should be examined in future research using established drug safety study methods. The majority of the clinics contributing to this EMR database are located in the southern US, limiting the geographic representativeness of the source population.

Despite the above limitations of the database, this data source is also an important strength of the present analysis. This study is based on data from outpatient oncology practices as opposed to the artificially constrained data obtained in clinical trials. Any patient with 1 of the 11 cancer types who did not have hypertension at baseline and who did have at least 1 BP measurement after the first cancer diagnosis was included in the study. No exclusions were made based on noncancer-related health status, age, or any of the other exclusions typically made in clinical trials. These data reflect real-world practice in oncology and the patterns of chemotherapy use and new-onset hypertension that occur.

## 5. Conclusions

HTN is a risk factor for coronary heart disease, stroke, heart failure, and end-stage renal disease, and the results of this study provide real-world data on the natural history of hypertension in cancer patients from US outpatient oncology practices. New-onset hypertension, regardless of severity, was observed in about one-third of cancer patients with various types of solid tumors. The incidence of hypertension at different severity levels varied with tumor type, and patients with gastric and ovarian cancer experienced the highest incidences of severe and crisis-level hypertension. Chemotherapy use appeared to be associated with an elevated risk of hypertension at each severity level, but blood pressure monitoring was more frequent during chemotherapy exposure.

## Figures and Tables

**Table 1:** ICD-9 diagnosis codes for cancer types of interest.

Cancer	ICD-9 diagnosis codes
Breast	174–174.9
Lung	162–162.9
Colorectal	153–154.8
Head and neck	141–141.9, 143–146.9, 148–149.9, 160–161.9
Gastric	151–151.9
Ovarian	183
Cervical	180–180.9
Renal	189.0, 189.1
Melanoma	172–172.9
Prostate	185
Connective and other soft tissue	171–171.9

ICD-9: International Classification of Diseases, 9th Revision.

**Table 2:** Demographics and baseline characteristics.

Patient characteristics	*N*, % *N* = 25090	Mean (SD) Median, range
Gender		
Male	8916 (35.5%)	
Female	16174 (64.5%)	
Age years		61.4 (13.36) 62.00, 18.00–99.00
Age categorized		
18–<30	208 (0.8%)	
30–<50	4770 (19.0%)	
50–<65	9436 (37.6%)	
≥65	10676 (42.6%)	
Year of index date		
2000–2002	2851 (11.4%)	
2003–2005	10793 (43.0%)	
2006–2008	11446 (45.6%)	
Clinic type		
Hospital	8313 (33.1%)	
Community	16777 (66.9%)	
Health insurance type		
Private	5528 (22.0%)	
Public	3169 (12.6%)	
Self	138 (0.6%)	
Mixed	5784 (23.1%)	
Other/unknown	10471 (41.7%)	
Region of residence		
Northeast	318 (1.3%)	
South	17203 (68.6%)	
Midwest	4218 (16.8%)	
West	3351 (13.4%)	
Cancer type		
Breast	9049 (36.1%)	
Lung	6434 (25.6%)	
Colorectal	4018 (16.0%)	
Head and neck	754 (3.0%)	
Gastric	386 (1.5%)	
Ovarian	763 (3.0%)	
Cervical	313 (1.2%)	
Renal	548 (2.2%)	
Melanoma	751 (3.0%)	
Prostate	1596 (6.4%)	
Connective and other soft tissue	478 (1.9%)	
Baseline SBP mm Hg		125.1 (14.39) 126.00, 64.00–150.00
Baseline SBP mm Hg		
<120	8089 (32.2%)	
120–50	17001 (67.8%)	
Baseline DBP mm Hg		74.3 (9.82) 74.00, 30.00–100.00
Baseline DBP mm Hg		
<80	16609 (66.2%)	
80–100	8481 (33.8%)	
Baseline BP status		
Ideal normal BP	7353 (29.3%)	
Prehypertension	17737 (70.7%)	

Ideal normal BP: SBP < 120 mm Hg and DBP < 80 mm Hg. Prehypertension: SBP 120–150 mm Hg or DBP 80–100 mm Hg. BP: blood pressure; DBP: diastolic blood pressure; *N*: number of patients; SBP: systolic blood pressure; SD: standard deviation.

**Table 3:** Time to first occurrence of hypertension.

	Moderate hypertension			Severe hypertension			Crisis-level hypertension		
	*N* with elevation	Median	Mean (SD)	*N* with elevation	Median	Mean (SD)	*N* with elevation	Median	Mean (SD)
Time to first occurrence of hypertension (days)									
Any hypertension	7420	96	231 (338)	3946	122	269 (362)	1003	183	341 (404)
SBP	7099	98	235 (344)	3877	121	269 (364)	961	181	342 (411)
DBP	1382	155	320 (399)	251	199	337 (348)	100	263	471 (536)
BP at first occurrence of hypertension (mm Hg)									
SBP	7099	155	155.3 (3.1)	3877	167	167.7 (5.5)	961	186	188.5 (7.6)
DBP	1382	103	104.2 (2.9)	251	114	114.5 (3.0)	100	128	131.5 (10.8)

Moderate hypertension: 150 mm Hg < SBP ≤ 160 mm Hg or 100 mm Hg < DBP ≤ 110 mm Hg. Severe hypertension: 160 mmHg < SBP ≤ 180 mm Hg or 110 mm Hg < DBP ≤ 120 mm Hg. Crisis-level hypertension: 180 mm Hg < SBP or 120 mm Hg < DBP. The arithmetic medians and means were calculated only among patients with the event. BP: blood pressure; CI: confidence interval; DBP: diastolic blood pressure; *N*: number of patients; SBP: systolic blood pressure; SD: standard deviation.

**Table 4:** Incidence of hypertension by cancer type and chemotherapy status.

	Number of patients evaluated	Incidence of hypertension					
		Moderate hypertension		Severe hypertension		Crisis-level hypertension	
		Number of patients with moderate hypertension	Incidence rate per 100 person-years (95% CI)	Number of patients with severe hypertension	Incidence rate per 100 person-years (95% CI)	Number of patients with crisis hypertension	Incidence rate per 100 person-years (95% CI)
Overall							
Total	25090	7420 (29.6%)	27.26 (18.00–39.59)	3946 (15.7%)	12.36 (6.46–21.42)	1003 (4.0%)	2.79 (0.53–8.45)
Cancer type							
Breast	9049	2565 (28.3%)	18.38 (10.96–28.91)	1319 (14.6%)	8.11 (3.53–15.91)	325 (3.6%)	1.80 (0.18–6.90)
Lung	6434	1799 (28.0%)	41.42 (29.78–56.11)	913 (14.2%)	17.95 (10.63–28.38)	234 (3.6%)	4.11 (1.14–10.39)
Colorectal	4018	1446 (36.0%)	35.60 (24.88–49.38)	830 (20.7%)	16.92 (9.84–27.12)	234 (5.8%)	4.10 (1.14–10.38)
Head and neck	754	191 (25.3%)	34.82 (24.23–48.46)	102 (13.5%)	16.43 (9.47–26.51)	25 (3.3%)	3.64 (0.91–9.72)
Gastric	386	95 (24.6%)	38.83 (27.59–53.12)	52 (13.5%)	18.45 (11.02–29.00)	18 (4.7%)	5.57 (1.95–12.47)
Ovarian	763	270 (35.4%)	37.76 (26.69–51.88)	173 (22.7%)	20.24 (12.40–31.18)	49 (6.4%)	4.79 (1.51–11.37)
Cervical	313	83 (26.5%)	34.17 (23.69–47.72)	44 (14.1%)	15.73 (8.94–25.65)	9 (2.9%)	2.96 (0.60–8.70)
Renal	548	184 (33.6%)	46.72 (34.29–62.18)	86 (15.7%)	17.01 (9.91–27.23)	21 (3.8%)	3.68 (0.93–9.78)
Melanoma	751	133 (17.7%)	16.67 (9.66–26.82)	64 (8.5%)	7.26 (2.98–14.77)	18 (2.4%)	1.92 (0.22–7.10)
Prostate	1596	537 (33.6%)	38.12 (26.99–52.30)	305 (19.1%)	18.00 (10.67–28.45)	61 (3.8%)	3.06 (0.65–8.86)
Connective and other soft tissue	478	117 (24.5%)	23.02 (14.60–34.54)	58 (12.1%)	10.29 (4.99–18.76)	9 (1.9%)	1.42 (0.09–6.29)

During chemotherapy							
Total	13167	3647 (27.7%)	90.07 (72.43–110.70)	1901 (14.4%)	40.21 (28.76–54.71)	478 (3.6%)	8.98 (4.10–17.06)
Cancer type							
Breast	4144	1034 (25.0%)	70.45 (54.97–88.95)	508 (12.3%)	30.04 (20.28–42.88)	112 (2.7%)	5.98 (2.19–13.04)
Lung	4294	1125 (26.2%)	102.02 (83.19–123.84)	563 (13.1%)	44.27 (32.20–59.37)	142 (3.3%)	10.15 (4.90–18.58)
Colorectal	2548	908 (35.6%)	104.50 (85.43–126.56)	515 (20.2%)	48.58 (35.89–64.30)	131 (5.1%)	10.48 (5.13–19.01)
Head and neck	402	93 (23.1%)	131.01 (109.53–155.46)	46 (11.4%)	57.17 (43.32–74.04)	16 (4.0%)	18.39 (10.97–28.93)
Gastric	190	37 (19.5%)	69.48 (54.11–87.86)	21 (11.1%)	36.80 (25.88–50.77)	10 (5.3%)	16.34 (9.40–26.41)
Ovarian	588	205 (34.9%)	93.52 (75.53–114.50)	124 (21.1%)	49.42 (36.61–65.26)	37 (6.3%)	12.38 (6.47–21.44)
Cervical	169	39 (23.1%)	129.39 (108.05–153.70)	21 (12.4%)	63.82 (49.13–81.52)	3 (1.8%)	7.80 (3.32–15.50)
Renal	172	46 (26.7%)	109.22 (89.70–131.73)	21 (12.2%)	38.41 (27.24–52.63)	11 (6.4%)	19.54 (11.86–30.33)
Melanoma	100	13 (13.0%)	59.05 (44.95–76.16)	8 (8.0%)	36.54 (25.67–50.47)	3 (3.0%)	12.51 (6.57–21.61)
Prostate	381	115 (30.2%)	94.43 (76.35–115.51)	62 (16.3%)	42.12 (30.37–56.90)	10 (2.6%)	5.78 (2.07–12.76)
Connective and other soft tissue	179	32 (17.9%)	63.40 (48.76–81.06)	12 (6.7%)	20.24 (12.40–31.17)	3 (1.7%)	4.78 (1.50–11.36)

During the period with no chemotherapy							
Total	22969	5057 (22.0%)	20.89 (12.92–31.97)	2621 (11.4%)	9.64 (4.55–17.92)	619 (2.7%)	2.09 (0.27–7.36)
Cancer type							
Breast	8660	1973 (22.8%)	15.09 (8.46–24.85)	985 (11.4%)	6.72 (2.64–14.04)	250 (2.9%)	1.58 (0.13–6.54)
Lung	5571	971 (17.4%)	28.77 (19.23–41.38)	489 (8.8%)	13.10 (7.00–22.36)	109 (2.0%)	2.70 (0.49–8.31)
Colorectal	3576	866 (24.2%)	25.20 (16.34–37.14)	478 (13.4%)	12.35 (6.45–21.40)	133 (3.7%)	3.11 (0.67–8.93)
Head and neck	673	118 (17.5%)	24.45 (15.74–36.25)	64 (9.5%)	12.24 (6.37–21.26)	9 (1.3%)	1.58 (0.13–6.55)
Gastric	343	69 (20.1%)	33.34 (22.99–46.74)	38 (11.1%)	17.27 (10.11–27.55)	9 (2.6%)	3.54 (0.87–9.58)
Ovarian	628	158 (25.2%)	29.67 (19.97–42.43)	98 (15.6%)	16.59 (9.59–26.71)	18 (2.9%)	2.73 (0.51–8.36)
Cervical	275	53 (19.3%)	25.17 (16.31–37.10)	28 (10.2%)	11.66 (5.96–20.53)	6 (2.2%)	2.36 (0.36–7.79)
Renal	520	153 (29.4%)	43.19 (31.28–58.14)	73 (14.0%)	16.37 (9.42–26.44)	10 (1.9%)	1.98 (0.24–7.20)
Melanoma	732	124 (16.9%)	16.08 (9.20–26.08)	60 (8.2%)	7.04 (2.84–14.48)	16 (2.2%)	1.77 (0.18–6.86)
Prostate	1554	475 (30.6%)	36.43 (25.58–50.34)	260 (16.7%)	16.77 (9.73–26.93)	52 (3.3%)	2.90 (0.58–8.62)
Connective and other soft tissue	437	97 (22.2%)	21.34 (13.27–32.51)	48 (11.0%)	9.59 (4.52–17.86)	7 (1.6%)	1.26 (0.06–6.01)

Moderate hypertension: 150 mm Hg < SBP ≤ 160 mm Hg or 100 mm Hg < DBP ≤ 110 mm Hg. Severe-hypertension: 160 mm Hg < SBP ≤ 180 mm Hg or 110 mm Hg < DBP ≤ 120 mm Hg. Crisis-level hypertension: 180 mm Hg < SBP or 120 mm Hg < DBP.

**Table 5:** Cox proportional hazards regression analysis identifying predictors of new-onset hypertension.

Baseline and clinical characteristics	Moderate or higher hypertension		Severe or crisis-level hypertension		Crisis-level hypertension	
	Hazard ratio (95% CI)	*P* value	Hazard ratio (95% CI)	*P* Value	Hazard ratio (95% CI)	*P* value
Gender						
Male	Reference		Reference		Reference	
Female	1.02 (0.97–1.09)	0.42	1.29 (1.09–1.53)	0.0027	1.28 (1.08–1.51)	0.0039
Age						
18–<30	0.60 (0.42–0.87)	0.0073	0.00 (0.00–>999)	0.92	0.00 (0.00–>999)	0.92
30–<50	Reference		Reference		Reference	
50–<65	1.55 (1.45–1.67)	<0.0001	1.87 (1.49–2.35)	<0.0001	1.87 (1.49–2.34)	<0.0001
≥65	2.13 (1.97–2.30)	<0.0001	2.71 (2.13–3.46)	<0.0001	2.74 (2.15–3.50)	<0.0001
Year of diagnosis						
2000–2002	0.95 (0.88–1.02)	0.16	1.20 (0.97–1.49)	0.096	0.98 (0.79–1.23)	0.89
2003–2005	0.93 (0.88–0.97)	0.0023	1.10 (0.95–1.26)	0.2	0.97 (0.84–1.12)	0.67
2006–2008	Reference		Reference		Reference	
Cancer type						
Breast	Reference		Reference		Reference	
Lung	1.20 (1.12–1.29)	<0.0001	1.25 (1.03–1.51)	0.027	1.38 (1.13–1.67)	0.0013
Colorectal	1.33 (1.24–1.43)	<0.0001	1.72 (1.42–2.08)	<0.0001	1.77 (1.46–2.14)	<0.0001
Head and neck	1.29 (1.11–1.49)	0.0007	1.55 (1.01–2.37)	0.044	1.74 (1.13–2.66)	0.011
Gastric	1.31 (1.08–1.60)	0.0069	2.14 (1.31–3.47)	0.0022	2.37 (1.46–3.85)	0.0005
Ovarian	1.31 (1.16–1.48)	<0.0001	1.89 (1.38–2.57)	<0.0001	1.95 (1.43–2.66)	<0.0001
Cervical	1.63 (1.33–2.00)	<0.0001	1.46 (0.75–2.85)	0.26	1.63 (0.84–3.18)	0.15
Renal	1.83 (1.57–2.12)	<0.0001	1.52 (0.96–2.42)	0.074	1.67 (1.05–2.66)	0.03
Melanoma	1.07 (0.90–1.26)	0.44	1.31 (0.81–2.13)	0.27	1.41 (0.86–2.29)	0.17
Prostate	1.47 (1.32–1.64)	<0.0001	1.47 (1.06–2.04)	0.021	1.54 (1.11–2.14)	0.0095
Connective and other soft tissue	1.32 (1.10–1.58)	0.0022	1.00 (0.51–1.95)	0.99	1.05 (0.54–2.05)	0.89
Baseline BP status						
Ideal normal BP	Reference		Reference		Reference	
Prehypertension	2.60 (2.45–2.77)	<0.0001	2.56 (2.13–3.07)	<0.0001	2.54 (2.12–3.05)	<0.0001
Clinic type						
Hospital	Reference		Reference		Reference	
Community	0.88 (0.83–0.93)	<0.0001	1.29 (1.09–1.52)	0.0026	1.24 (1.05–1.46)	0.01
Health insurance type						
Private	Reference		Reference		Reference	
Public	1.18 (1.08–1.28)	0.0002	1.45 (1.12–1.86)	0.0042	1.47 (1.14–1.89)	0.0032
Self	1.41 (1.02–1.94)	0.035	0.38 (0.05–2.69)	0.33	0.39 (0.05–2.81)	0.35
Mixed	1.09 (1.01–1.18)	0.026	1.42 (1.12–1.79)	0.0034	1.41 (1.12–1.78)	0.0037
Other/unknown	1.09 (1.03–1.16)	0.0057	1.64 (1.35–1.99)	<0.0001	1.61 (1.33–1.95)	<0.0001
Region of residence						
Northeast	Reference		Reference		Reference	
South	1.26 (1.00–1.58)	0.049	1.75 (0.83–3.70)	0.14	1.68 (0.79–3.55)	0.18
Midwest	1.56 (1.23–1.97)	0.0002	2.54 (1.19–5.43)	0.016	2.42 (1.13–5.17)	0.022
West	0.85 (0.67–1.09)	0.2	1.13 (0.52–2.47)	0.75	1.10 (0.51–2.40)	0.81
Chemotherapy						
No chemotherapy	Reference		Reference		Reference	
First-line cytotoxic	2.19 (2.07–2.32)	<0.0001	1.98 (1.69–2.33)	<0.0001	2.65 (2.24–3.14)	<0.0001
First-line targeted	2.28 (1.90–2.74)	<0.0001	2.99 (1.89–4.73)	<0.0001	3.45 (2.18–5.46)	<0.0001
First-line combination	3.39 (3.05–3.76)	<0.0001	3.20 (2.44–4.20)	<0.0001	3.65 (2.78–4.80)	<0.0001
Second-line cytotoxic	2.46 (2.18–2.76)	<0.0001	1.66 (1.11–2.49)	0.013	1.56 (1.04–2.33)	0.03
Second-line targeted	3.15 (1.78–5.57)	<0.0001	7.93 (2.52–24.93)	0.0004	6.69 (2.13–21.04)	0.0011
Second-line combination	3.52 (3.07–4.05)	<0.0001	2.79 (1.81–4.31)	<0.0001	2.45 (1.59–3.78)	<0.0001
Third-line cytotoxic	2.77 (2.18–3.54)	<0.0001	1.60 (0.59–4.30)	0.35	1.22 (0.45–3.28)	0.69
Third-line targeted	3.49 (1.45–8.36)	0.0052	8.01 (1.12–57.31)	0.038	5.44 (0.76–38.88)	0.091
Third-line combination	2.50 (1.97–3.17)	<0.0001	1.57 (0.58–4.21)	0.37	1.16 (0.43–3.11)	0.77

Ideal normal BP: SBP < 120 mm Hg and DBP < 80 mm Hg. Prehypertension: SBP 120–150 mm Hg or DBP 80–100 mm Hg. Moderate hypertension: 150 mm Hg < SBP ≤ 160 mm Hg or 100 mm Hg < DBP ≤ 110 mm Hg. Severe hypertension: 160 mm Hg < SBP ≤ 180 mm Hg or 110 mm Hg < DBP ≤ 120 mm Hg. Crisis-level hypertension: 180 mm Hg < SBP or 120 mm Hg < DBP. BP: blood pressure; CI: confidence interval; DBP: diastolic blood pressure; SBP: systolic blood pressure.
